# Data for GAW20: genome-wide DNA sequence variation and epigenome-wide DNA methylation before and after fenofibrate treatment in a family study of metabolic phenotypes

**DOI:** 10.1186/s12919-018-0114-0

**Published:** 2018-09-17

**Authors:** Stella Aslibekyan, Laura Almasy, Michael A. Province, Devin M. Absher, Donna K. Arnett

**Affiliations:** 1 Department of Epidemiology, School of Public Health, University of Alabama at Birmingham, 1665 University Blvd, Birmingham, AL 35205 USA; 2 Department of Biomedical and Health Informatics, Children’s Hospital of Philadelphia, 3401 Civic Center Blvd, Philadelphia, PA 19104 USA; 3 Division of Statistical Genomics, Washington University in St Louis, 660 South Euclid Ave, St Louis, MO 63110 USA; 4 Hudson Alpha Institute for Biotechnology, 601 Genome Way, Huntsville, AL 35806 USA; 5 College of Public Health, University of Kentucky, 111 Washington Ave, Lexington, KY 40536 USA

## Abstract

GAW20 provided participants with an opportunity to comprehensively examine genetic and epigenetic variation among related individuals in the context of drug treatment response. GAW20 used data from 188 families (*N* = 1105) participating in the Genetics of Lipid Lowering Drugs and Diet Network (GOLDN) study (clinicaltrials.gov identifier NCT00083369), which included CD4+ T-cell DNA methylation at 463,995 cytosine-phosphate-guanine (CpG) sites measured before and after a 3-week treatment with fenofibrate, single-nucleotide variation at 906,600 loci, metabolic syndrome components ascertained before and after the drug intervention, and relevant covariates. All GOLDN participants were of European descent, with an average age of 48 years. In addition, approximately half were women and approximately 40% met the diagnostic criteria for metabolic syndrome. Unique advantages of the GAW20data set included longitudinal (3 weeks apart) measurements of DNA methylation, the opportunity to explore the contributions of both genotype and DNA methylation to the interindividual variability in drug treatment response, and the familial relationships between study participants. The principal disadvantage of GAW20/GOLDN data was the spurious correlation between batch effects and fenofibrate effects on methylation, which arose because the pre- and posttreatment methylation data were generated and normalized separately, and any attempts to remove time-dependent technical artifacts would also remove biologically meaningful changes brought on by fenofibrate. Despite this limitation, the GAW20 data set offered informative, multilayered omics data collected in a large population-based study of common disease traits, which resulted in creative approaches to integration and analysis of inherited human variation.

## Background

Epigenetic processes, defined as non–sequence-dependent heritable changes in gene expression [1], play a critical role in human development and disease. Broadly, epigenetic modifications include DNA methylation, histone modifications, and RNA-based mechanisms, as well as their interactions. Of these, DNA methylation is the most studied and measured in epidemiologic cohorts. With the advent of array technology enabling methylome-wide profiling at single-nucleotide resolution [2], numerous studies have identified and validated tissue-specific methylation patterns associated with aging [3], disease states (eg, cancer, obesity, autoimmune disease, among many others) [4–6], health behaviors (eg, diet, smoking, and alcohol intake) [7–9], environmental conditions (eg, air pollution and socioeconomic adversity) [10, 11], and other complex traits. Epigenetic processes such as DNA methylation reflect the dual influence of the underlying genomic sequence and the environment [12], linking innate predisposition with modifiable risk factors, altering downstream gene expression, and providing a plausible mechanism for disease pathogenesis. The DNA methylation “signature” can be both stable [13] and dynamic [14] over time, although the temporal component of epigenomic variation remains understudied owing to the limited availability of longitudinal measurements in large cohorts, as well as of appropriate statistical methods.

Because of the well-documented influence of sequence mutations on epigenetic patterns [15], it is prudent for association studies to examine both types of variation, capturing the trait architecture more completely. This general approach, which encompasses methylation quantitative trait loci (meQTL) analysis, Mendelian randomization with meQTL as the instrument, and other integrative techniques, has been successfully implemented in several genome- and epigenome-wide analyses of complex traits, such as glucose metabolism markers [16], blood lipids [17], schizophrenia [18], and obesity [19]. One notable exception is the area of pharmacogenetics, which has largely focused on contributions of DNA sequence variants, although the biological plausibility of links between methylation and drug response has been known for decades [20]. DNA methylation may serve as both the determinant of drug effects (upstream) and their modifier (downstream). For example, methyl conjugation is a prominent mechanism of drug metabolism, and the activities of the relevant enzymes may be affected by upstream DNA sequence mutations [20]. Despite evidence in support of these complex relationships and their translational promise, few studies to date have used both genetic and epigenetic variants to predict treatment response, and even fewer pharmacoepigenetic findings have been implemented in the clinic [21].

Recognizing the powerful promise of integrative pharmacogenomic research to advance the current understanding of complex traits, the GAW20 analyzed a family-based data set that includes epigenome-wide DNA methylation measurements at > 450,000 cytosine-phosphate-guanine (CpG) sites in CD4+ T cells before and after a pharmaceutical intervention, and genome-wide sequence variation at 718,542 unique single-nucleotide polymorphisms (SNPs). Other included covariates were sex, age, study site (Minnesota or Utah), smoking, and metabolic phenotypes (fasting triglycerides [TGs] and high-density lipoprotein [HDL] cholesterol before and after treatment, plus metabolic syndrome diagnosis). In addition to these real data, the GAW20 data distribution also included 200 replicates of simulated posttreatment methylation and TG measurements.

## Methods

### The GOLDN study

The Genetics of Lipid Lowering Drugs and Diet Network (GOLDN) study (clinicaltrials.gov identifier NCT00083369) was designed to evaluate genetic contributions to lipid response to lipid-raising and lipid-lowering interventions: a high-fat milkshake challenge to raise plasma TGs and a 3-week daily treatment with 160 mg of micronized fenofibrate, respectively. Participants, who self-reported as being predominantly of European descent, were recruited in 2002–2004 from 3-generational families previously screened at the Minnesota and Utah centers of the National Heart, Lung, and Blood Institute Family Heart Study [22]. Participants were eligible for screening if they were 18 years of age or older, and came from a family with at least 2 members in a sibship. Approximately 1350 individuals were screened and the following exclusion criteria were applied: fasting TGs ≥1500 mg/dL; recent history of myocardial infarction or *revascularization*; history of liver, kidney, pancreas, or gallbladder disease (including abnormal liver function tests and creatinine levels > 2.0 mg/dL); history of nutrient malabsorption; current use of insulin; or currently pregnant, breastfeeding, or not using an hormonal or barrier form of contraception for women of childbearing potential.

**Fig. 1:**
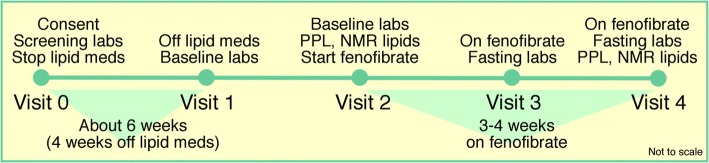
Sequence and timing of interventions and clinic visits for the GOLDN study (not to scale). NMR, nuclear magnetic resonance; PPL, postprandial lipemia

Following the eligibility screening, participants were asked to consult their physician and to provide informed consent to discontinue lipid-lowering drugs or dietary supplements for at least 4 weeks prior to the study. Figure 1 illustrates the sequence of interventions. During the first intervention (postprandial lipemia, visit 2), which occurred approximately 1 day after the baseline visit, participants were offered a high-fat meal (a flavored milkshake with 83% calories from fat, 700 cal/m^2^) and blood samples were drawn at 0, 3.5, and 6 h following meal ingestion. The GAW20 data set focuses on data from the second intervention, an approximately 3-week open-label trial of 160 mg fenofibrate taken daily beginning at visit 2 and ending at visit 3. The high-fat meal was repeated at visit 4 (approximately 1 day after visit 3), upon completion of the fenofibrate intervention. Participants were given the option to complete either or both interventions. The GAW20 data set contained pre- (visit 2) and post-fenofibrate (visit 4) measurements of epigenome-wide DNA methylation, genotype, lipid profile (specifically fasting TGs and HDL cholesterol from all 4 visits), metabolic syndrome diagnosis, and relevant covariates. If not specified otherwise, the included data were collected during visit 2.

### Phenotype measurements

Participants were asked to fast for at least 12 h prior to each study visit. TGs were measured by glycerol blanked enzymatic method using the COBAS FARA centrifugal analyzer (Roche Diagnostics); HDL-cholesterol was measured using the Roche/Hitachi 911 Automatic Analyzer (Roche Diagnostics) via a cholesterol esterase/cholesterol oxidase reaction [23]. All plasma samples were analyzed together at the end of the study. Age and smoking (current, past, or never) were ascertained via self-report. Metabolic syndrome was defined using 2 sets of criteria, provided by the National Cholesterol Education Program Adult Treatment Panel III (NCEP/ATP) [24] and the International Diabetes Federation (IDF) [25] (Table 1).

**Table 1:** Comparison of the IDF and the NCEP/ATP definitions of metabolic syndrome

NCEP/ATP	IDF
3 or more of the following: 1. Abdominal obesity: waist circumference ≥ 102 cm (men),≥88 cm (women) 2. Hypertriglyceridemia: TGs ≥ 150 mg/dL 3. Low HDL cholesterol: < 40 mg/dL (men), < 50 mg/dL (women) 4. High blood pressure: > 130/85 mmHg 5. High fasting glucose: > 110 mg/dL	Abdominal obesity as defined by the NCEP/ATP plus 2 or more of the following: 1. Hypertriglyceridemia: TGs ≥ 150 mg/dL or history of treatment 2. Low HDL cholesterol: < 40 mg/dL (men), < 50 mg/dL (women), or history of treatment 3. High blood pressure: diastolic ≥130 mmHg or systolic ≥85 mmHg or history of treatment 4. High fasting glucose: > 100 mg/dL or previous diagnosis of diabetes

To ascertain specific components of the metabolic syndrome (not included in the GAW20 release), the following measurements were performed in addition to the lipid assays described above: waist circumference over the unclothed abdomen at the umbilicus at the end of a normal expiration, blood pressure with an automated oscillometric device in a seated position after 5 min of rest, and fasting glucose with a hexokinase-mediate reaction using the Roche/Hitachi 911 Automatic Analyzer (Roche Diagnostics) [26]. Table 2 outlines the phenotypic summary of GAW20 participants. Participants spanned 188 families (mean family size = 5.9; SD = 4.7; median = 4.5; interquartile range [IQR] = 3.0–7.0).

**Table 2:** Demographic and clinical characteristics of GAW20 participants

	Mean/median (SD/IQR)	N
Female, n (%)	576 (52.1)	1105
Age, years^a^	48.2 (16.3)	1105
Center, n (%):		1105
Minnesota	565 (51.1)	
Utah	540 (48.9)	
TGsvisit 1, mg/dL^b^	119 (79;180)	802
TGsvisit 2, mg/dL^b^	109 (73;171)	1103
TGsvisit 3, mg/dL^b^	75.0 (53;112)	625
TGsvisit 4, mg/dL^b^	75.0 (53;108)	818
HDL-cholesterol visit 1, mg/dL^a^	46.5 (13.2)	802
HDL-cholesterol visit 2, mg/dL^a^	47.2 (13.1)	1104
HDL-cholesterol visit 3, mg/dL^a^	49.3 (13.4)	793
HDL-cholesterol visit 4, mg/dL^a^	49.4 (13.5)	861
Smoking, n (%):		1104
Never smoker	780 (70.7)	
Past smoker	239 (21.6)	
Current smoker	85 (7.7)	
NCEP/ATP diagnosis of metabolic syndrome, n (%)	418 (37.8)	1105
IDF diagnosis of metabolic syndrome, n (%)	458 (41.4)	1105

^a^Mean (SD) ^b^Median (IQR)

### DNA methylation measurements and quality control

DNA methylation data at the pre- and post-fenofibrate time points were generated 1 year apart. CD4+ T cells were isolated using antibody-linked magnetic beads (Invitrogen) from frozen buffy coat samples according to the manufacturer’s protocol. Cells that were captured on the beads were then lysed and DNA was extracted using the DNeasy Kit (Qiagen). The Infinium Human Methylation 450 K BeadChip (Illumina) was used to quantify epigenome-wide methylation. Following the standard steps of bisulfite treatment, amplification, hybridization, and imaging, intensity files were analyzed with Illumina Genome Studio software, which provided beta scores (the proportion of total signal from the methylation-specific probe) and detection *p* values (1 minus the probability that the target signal was distinguishable from negative control). All the steps described above were performed on pre- and post-fenofibrate data separately; quality control was conducted on pre- and post-fenofibrate data together, as follows. Beta scores for CpG sites where detection *p* value was> 0.01 or more than 10% of samples failed to yield adequate intensity were removed, as were samples with more than 1.5% missing data points. The final quality control (QC) step eliminated any CpG sites where the probe sequence mapped either to a location that did not match the annotation file or to > 1 locus. Such markers were identified by realigning all probes (with unconverted Cs) to the human reference genome. Following QC, there were methylation data from 463,995 CpGs, including those where methylation may be influenced by a SNP on the probe. Principal components based on the beta values of all autosomal CpG sites passing QC were generated by using the *prcomp* function in R and used to adjust for cell purity in association analysis [27]. As a result of the QC procedures described above, a small number of participants have measurements at either pre- or posttreatment visit, but not both.

### Batch effects

There were 3 main sources of technical variation in the GAW20 methylation data: (a) array-to-array variation (12 sample groups), (b) bisulfite conversion batches, and (c) changes (ie, a linear degradation) in the performance of the Illumina scanner laser over time. In GOLDN, the pretreatment samples were all run a year earlier than the posttreatment samples, and likely carry batch effects of all 3 types.

We could control the array-to-array variation and the bisulfite conversion batches within the pre- and posttreatment groups with ComBat [28] normalization. Data from pre- and posttreatment methylation measurements were normalized at the same time with the same software in 2 separate batches, as follows. Within each treatment group (ie, pre- or post-fenofibrate), all beta scores for CpGs that passed QC were normalized using ComBat with randomly selected subsets of 20,000 CpGs per run, each array of 12 samples as a “batch,” and adjustments for both plate and position within. Because the array groups are perfect subsets of the bisulfite conversion batches, such normalization corrects for batch effects of types (a) and (b) within each treatment group. Probes from Infinium I versus Infinium II chemistries present on each array were normalized separately, and beta scores from Infinium II probes were adjusted using a previously published equation [29].

However, the treatment status (pre- or post-fenofibrate) was perfectly correlated with the time when the data were generated. Therefore, any methylation changes observed from pre- to posttreatment represent both the effects of fenofibrate and processing time batch effects (ie, scanner drift). To avoid erasing treatment effects, all normalization took place within each treatment group (ie, pre- and post-fenofibrate treatment data were not normalized together) as described above. As a result, longitudinal comparisons of epigenetic data are affected by batch effects of all three types, whereas batch effects in cross-sectional analyses were removed through ComBat normalization.

### Genotyping

Genomic DNA was extracted from blood samples and purified using Puregene (Gentra System) according to the manufacturer’s protocol [30] in 842 GOLDN participants. Genotype at 906,600 (869,161 autosomal) loci was ascertained using the Affymetrix Genome Wide Human SNP Array 6.0 (Affymetrix) and the Birdseed algorithm; calling was performed in batches [31]. After removing monomorphic SNPs (55,530), SNPs with call rate < 96% (82,462), and Mendel errors (12,627), 718,542 unique autosomal SNPs (and 2 duplicated SNPs, chr2: rs1462062 and chr3: rs12635398) were available for further analyses. Additionally, 16 participants with call rate < 96% and 4 duplicates were removed, and 7 sample switches were corrected, with 822 genotyped individuals remaining in the data set after all QC procedures. To limit file size and facilitate data distribution, the GAW20 data set did not include imputed genotypes.

## Results and discussion

In the spirit of omics integration implemented in the GAW19 data set, which contained whole genome sequencing and transcriptomic data [32], GAW20 offered opportunities to jointly examine comprehensive patterns of DNA methylation and sequence variation in a family-based study. Further analytic opportunities were presented by repeated measurements of both DNA methylation and lipid outcomes (before and after the fenofibrate intervention), as well as by the lipid-lowering treatment itself, which opened the door for pharmacogenomic investigations. In addition to the real data from the pharmaceutical intervention, GAW20 included a companion problem aimed at discovering associations between methylation variants and TG response to a fictional drug using simulated data from 200 independent replications.

By measuring multilayered omics and phenotypic characteristics on the same participants, the GAW20 data set provided a springboard to develop much-needed methods for integrating methylation, genomic, and pharmacoepidemiologic data in large studies. To date, the increasing availability of high-dimensional omics data has outpaced the evolution of analytic tools, limiting discovery and translational applications [33]. Integrated analyses are poised to better reflect the underlying biological architecture of complex traits and in some cases strengthen causal inference [34]. However, multidimensional data pose a number of challenges related to statistical power and/or multiple testing burden, correlations between omic layers, confounding, and other method-specific emergent issues [33]. As a result, the optimal method for integrated analysis is likely to vary by research question and data structure; by standardizing the latter, GAW20 enabled the creativity of the former, supporting diverse approaches and solutions to common challenges.

Even though the GOLDN data set came with unique advantages, including longitudinal measurements and well-characterized genomic and epigenomic variation, certain features of the GAW20 data deserve further consideration. First, DNA methylation was quantified on CD4+ T cells, the most abundant lymphocyte in whole blood. This choice leveraged an easily accessible tissue as well as addressed the possibility of confounding by cell type, but may have muted the biological relevance of any findings resulting from the tissue-specific nature of DNA methylation [35]. Second, all GOLDN participants were of European American descent, which limited both confounding by population stratification and generalizability to other ethnic groups. Third, the methylation data had already been normalized, restricting the participants from improving current approaches to batch effects and probe chemistry corrections. Furthermore, because normalization on pre- and post-fenofibrate batches of samples was performed separately in GOLDN, investigations of longitudinal changes in methylation were hampered by the inextricable correlation between batch effects and fenofibrate effects on methylation. However, batch effects in cross-sectional analyses of methylation data and metabolic phenotypes were successfully removed using ComBat normalization, and other approaches to the batch effects problem were explored during the GAW20 workshop [36–38], offering possible solutions to future studies of epigenetic variation.

## Conclusions

The GAW20 data set provided a rich environment for timely methodological exploration at the intersection of DNA sequence, methylation, and complex traits, including response to drug treatment.
